# Supplementary figures and images for: Identification of small molecule agonists of fetal hemoglobin expression for the treatment of sickle cell disease
Source: PLoS One. 2024 Nov 6;19(11):e0307049. doi: 10.1371/journal.pone.0307049 (PMC11540224; doi:10.1371/journal.pone.0307049)

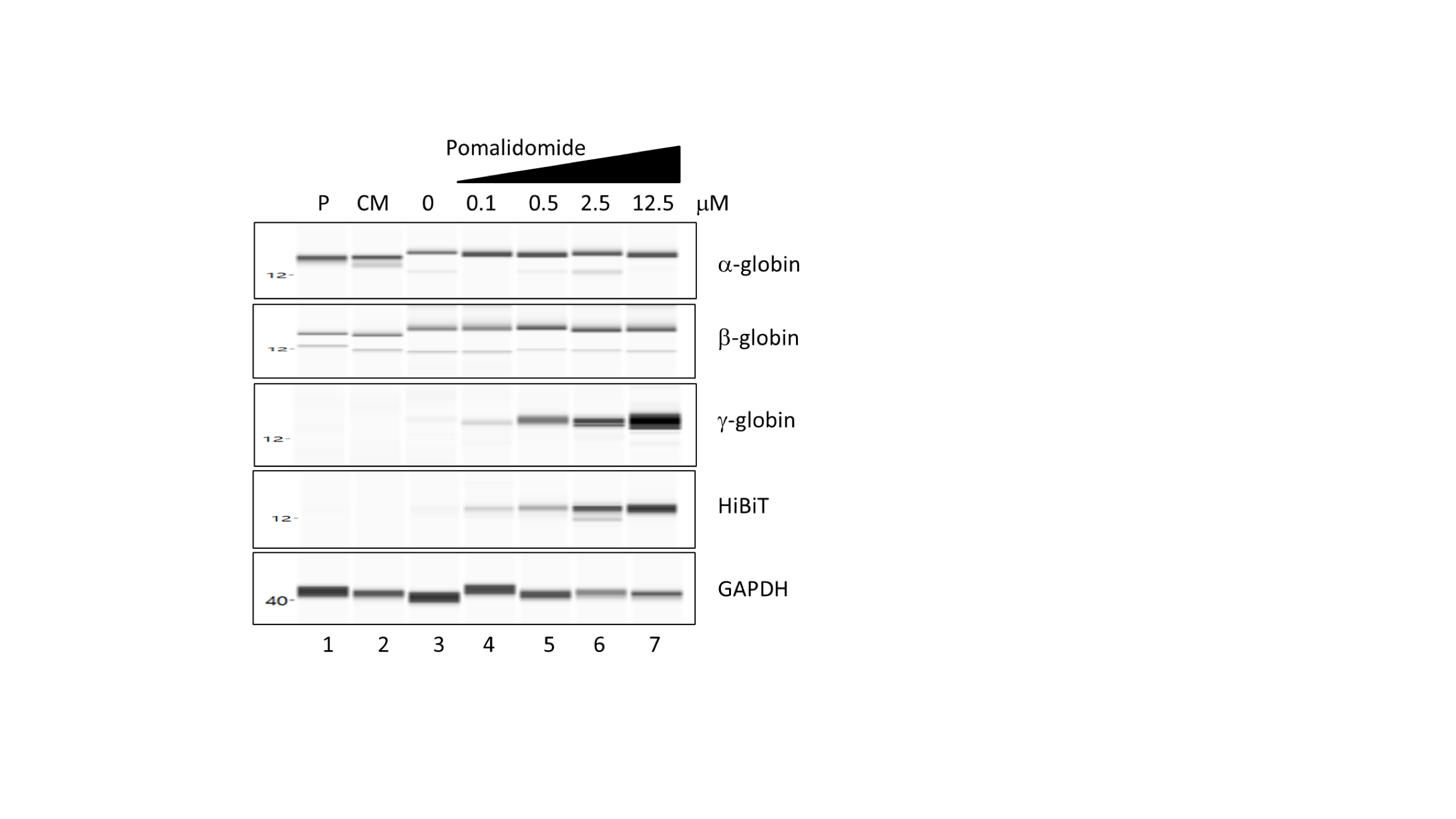

Supplement: S1 Fig — GAPDH was used as the loading control. Lane 1 (P): parental HUDEP2 cells; Lane 2 (CM): HUDEP2_HBG1_HiBiT cells cultured in expansion media; Lane 3: HUDEP2_HBG1_HiBiT cells in differentiation media with 0.05% DMSO and without pomalidomide. Lane 4–7: HUDEP2_HBG1_HiBiT cells in differentiation media with various concentrations of pomalidomide. (TIFF) [file pone.0307049.s001.tiff]
