# Supplementary material for: Identification of small molecule agonists of fetal hemoglobin expression for the treatment of sickle cell disease
Source: PLoS One. 2024 Nov 6;19(11):e0307049. doi: 10.1371/journal.pone.0307049 (PMC11540224; doi:10.1371/journal.pone.0307049)
Supplement: Attachment — Submitted filename: Response to Reviewers.docx [file pone.0307049.s006.docx]

5. Review Comments to the Author

Please use the space provided to explain your answers to the questions above. You may also include additional comments for the author, including concerns about dual publication, research ethics, or publication ethics. (Please upload your review as an attachment if it exceeds 20,000 characters)

**Reviewer #1:** This manuscript describes a new screening system for HbF-inducing drugs. There is a great need for these drugs because gene therapy and BM transplantation can only be used to treat a relatively small number of SCD and beta-thal patients. Four new HbF-inducers were identified. It will be of great interest to determine whether these drugs are active in vivo in SCD mice and nonhuman primate studies.

*The authors thank the reviewer for their kind comments and agree that future SCD mice studies and NHP models will be exciting to test these new therapies.*

**Reviewer #2:** A novel approach to addressing the issue of discovering new and/or existing therapeutics to induce fetal hemoglobin as a treatment option for individuals with sickle cell disease. Methods are clearly stated and easily reproducible/replicated—utilization of CRISPR and modern methods to accomplish goals laid out in the manuscript.

Minor Comments
- Line 257: add brief sentence about why pom was chosen as positive control over other agents

*We thank the reviewer for pointing out the reason for using pomlidamide as a positive control and have added a sentence at line 255 that describes it.*

- Please proofread again; minor errors were found
o Line 66 misspelling of azacytidine

*Spelling corrected.*

o Line 196 incorrect capitalization use in the word “complete”

*Incorrect capitalization corrected.*

**Reviewer #3:** hydoxyurea is the only drug approved for sickle cell disease which induces fetal hemoglobin. The author and colleagues identified small molecules that also induce fetal hemoglobin in cell lines. This is a study that is needed and is valuable and we need more drugs that can be used to help our patients with sickle cell disease.

*The authors thank the reviewer for that insightful point about hydroxyurea and agree wholeheartedly that new small molecule therapies are needed to treat this horrible disease.*

**In response to the journal request for gel blot uncropped original images:**

*The San Diego site of Takeda unfortunately shut its doors in August and all access to data was shut down. Consequently, the authors were unable to obtain the original western blot images for Fig 2E. Therefore, we have moved Fig 2E to Supplementary Fig 1 and we hope that will suffice for publication. If the original uncropped images for this new supplementary figure are required for publication, then we can omit the figure entirely without affecting the supporting data or flow of the paper. Please let us know how you would like to proceed.*

*Thank you,*

*-Shane Horman*
